# Pore-Scale Modeling of the Effect of Wettability on Two-Phase Flow Properties for Newtonian and Non-Newtonian Fluids

**DOI:** 10.3390/polym12122832

**Published:** 2020-11-28

**Authors:** Moussa Tembely, Waleed S. Alameri, Ali M. AlSumaiti, Mohamed S. Jouini

**Affiliations:** The Petroleum Institute, Khalifa University, Abu Dhabi P.O. Box 2533, UAE; walameri@pi.ac.ae (W.S.A.); aalsumaiti@pi.ac.ae (A.M.A.); mjouini@pi.ac.ae (M.S.J.)

**Keywords:** multiphase flow, volume of fluid method, wettability, non-Newtonian fluid

## Abstract

The Darcy-scale properties of reservoir rocks, such as capillary pressure and relative permeability, are controlled by multiphase flow properties at the pore scale. In the present paper, we implement a volume of fluid (VOF) method coupled with a physically based dynamic contact angle to perform pore-scale simulation of two-phase flow within a porous medium. The numerical model is based on the resolution of the Navier–Stokes equations as well as a phase fraction equation incorporating a dynamic contact angle model with wetting hysteresis effect. After the model is validated for a single phase, a two-phase flow simulation is performed on both a Newtonian and a non-Newtonian fluid; the latter consists of a polymer solution displaying a shear-thinning power law viscosity. To investigate the effects of contact angle hysteresis and the non-Newtonian nature of the fluid, simulations of both drainage and imbibition are carried out in order to analyze water and oil saturation—particularly critical parameters such as initial water saturation ($S_{wi}$) and residual oil saturation ($S_{or}$) are assessed in terms of wettability. Additionally, the model sensitivities to the consistency factor (χ), the flow behavior index (*n*), and the advancing and receding contact angles are tested. Interestingly, the model correctly retrieves the variation in $S_{or}$ and wettability and predicts behavior over a wide range of contact angles that are difficult to probe experimentally.

## 1. Introduction

Predicting multiphase properties of porous media is critical in many applications including the oil and gas industry, CO_2_ sequestration, water management, and environmental clean-up technology. Despite the large body of existing research, very few publications address the crucial role played by the wettability, quantified by the contact angle and its hysteresis, at the pore scale on the properties of both Newtonian and non-Newtonian fluids, which ultimately control fluid flow behavior at the Darcy scale. While the prediction of petrophysical properties by digital rock physics (DRP) [1,2,3,4] has gained attention recently, most of these simulations focus on Newtonian fluids and do not address either the detailed description of fluid–rock interaction or fluid rheological behavior, which can be non-Newtonian in many petroleum engineering applications, including heavy crude oil or polymer solutions used for enhanced oil recovery (EOR).

Understanding the effects of wettability in porous media is the subject of ongoing research. It is still very challenging to experimentally characterize the wettability of an oil/water/rock system in which the fluid distribution cannot be visualized and assessed accurately [5,6]. To overcome the experimental limitations, numerical approaches are often used to evaluate flow behavior at the pore scale. The complexities of the pore space microstructure and two-phase flow simulations make computation of flow properties very challenging. The numerical techniques used to conduct pore-scale simulations can be classified into two categories: (i) pore-network modeling [7] and (ii) direct modeling, which includes the finite difference method [8], the finite element method [9], the finite volume method [10,11], and the lattice Boltzmann method (LBM) [1]. The pore-network model (PNM) has been preferred over direct simulation to predict petrophysical properties due to its simplicity. In this approach, the pore space is represented by a simplifying geometric primitive consisting of the interconnections between spherical and cylindrical elements. The fluid flow through the network is described by an analytical relation between the different pores and throats. One of the limitations of PNM is its lack of generalizability as it requires adjusting many parameters in order to retrieve the observed experimental behavior. In addition, this approach cannot readily accommodate viscous and capillary forces while maintaining the geometry of the pore space. However, recently, we proposed through deep learning and LBM an improvement of PNM for a single phase flow [12].

In [13], a PNM is used to investigate the relative permeability and capillary pressure of a Berea sandstone. However, the fluid is Newtonian and the simulations are performed using a simplified pore space. While non-Newtonian fluid flow based on PNM has been investigated in [14,15], the dynamical nature of the contact angle has not been addressed. In order to gain meaningful physical insight into the flow behavior, this study adopts direct modeling, after demonstrating the limitations of PNM. Recently, direct pore-scale simulation of single-phase flow addressing the computation of the permeability has been performed for both Newtonian and non-Newtonian fluids [11,16,17]. An interesting experimental in situ assessment of static contact angles at the pore scale has been performed in [18] with Newtonian fluids. Recently, Xie et al. [19] developed an LBM method for modeling multiphase flow, and the model has been applied in [20] to assess the influence of shear-thinning or shear-thickening fluids on EOR. However, the model does not account for the critical effect of contact hysteresis on two-phase flow behavior.

Most of the two-phase flow models in the literature focus primarily on Newtonian fluid flow, assuming the contact angle to be static and not considering either its dynamics or hysteresis [17,19]. While a moving contact line is crucial for accurately describing multiphase flow behavior, modeling this moving contact line is still very challenging in situations where the fluid–solid interaction is controlled by the interplay between the advancing, receding, and static contact angles. The accuracy of the two-phase flow is determined by how the dynamic contact angle is modeled. Unlike in previous direct modeling studies in the literature, which use only the static contact angle, here we adopted a dynamic contact angle based on a modified Kistler’s correlation [21]. This is one of the most accurate methods for modeling the dynamic contact angle, taking as inputs the static, advancing, and receding contact angles between rock and fluid. We implemented a dynamic contact angle which accounts for these angles, while the phase fraction of the two-phase flow is modeled by the volume of fluid (VOF) method. To our knowledge, there has been very little previous numerical work addressing this aspect of pore-scale modeling.

In addition, fluid flow through porous media becomes even more complex if one of the fluids is non-Newtonian, as is the case in situations such as polymer flooding or heavy oil production. The goal of the present work is two-fold: (i) the development of an accurate model for simulating wettability at the pore scale with wetting hysteresis effect, which depends on the displacement direction (drainage or imbibition), and (ii) extension to non-Newtonian fluid flow through porous media, where the effect of fluid rheology on the two-phase flow properties can be assessed.

The novel contribution of this work is the development of a new simulator capable of computing the dynamics of two-phase Newtonian and non-Newtonian fluids and their interactions with rock using a dynamic contact angle model. The simulator, which can be used for different rock types at the pore scale, can provide valuable insight into the prediction of multiphase flow behavior in subsurface flow, such as in soil remediation, and can also provide better strategies for EOR.

The paper is organized as follows: Section 2 details the methodology and governing equations to address the modeling of both Newtonian and non-Newtonian fluids. The model validation and its application to porous media are provided in Section 3. Finally, conclusions are drawn in Section 4.

## 2. Governing Equations and Methodology

### 2.1. Two-Phase Flow Governing Equations

A numerical method based on the volume of fluid (VOF) method is adopted to accurately describe the fluid flow behavior within a porous medium at pore scale. The continuity and momentum equations to be numerically solved within VOF framework are expressed as follows:

mass conservation

(1) ∇.V=0,

momentum conservation

(2) $$\frac{\partial (\rho V)}{\partial t}+\nabla .\left(\rho \mathrm{VV}\right)=-\nabla p+\rho g+\nabla .\tau +\sigma \kappa \nabla \alpha ,$$

where V is the velocity vector, *p* is the pressure, σ is the surface tension, κ is the interface curvature, α is the phase fraction, τ is the stress tensor, and g is the gravity acceleration. The capillary effect is modeled as a force using the continuum surface force (CSF) approach [22], where the mean curvature (κ) in Equation (2) expresses as follows:

(3) $$\kappa =-\nabla .\left(\frac{\nabla \alpha }{\left|\alpha \right|}\right)$$

The two phases, oil and water, are defined through the phase fraction (α), which is advected by the flow following the transport equation using the interface compression method:

(4) $$\frac{\partial \alpha }{\partial t}+V\nabla \alpha +\nabla .\left[V_{c}\alpha \left(1-\alpha \right)\right]=0$$

The compression velocity $V_{c}$ describes the relative velocity at the free surface and is given by [23]:

(5) $$V_{c}=n_{f}\min\left[C_{\alpha }\frac{\phi_{f}}{S_{f}},\max\left(\frac{\phi_{f}}{S_{f}}\right)\right]$$

where $S_{f}$ represents the cell surface area through which a mass flux, $\phi_{f}$, flows. The parameter $C_{\alpha }$ is introduced to control the degree of the interface sharpness. In the present case, $C_{\alpha }$ = 1, which leads to a conservative compression [23,24]. Unlike the conventional VOF method, the interface compression scheme can be applied to complex unstructured meshes without the need for geometric algorithms for interface reconstruction. The compression term in Equation (5) acts only at the interface to suppress numerical diffusion and maintain the interface sharpness.

Finally, the physical properties in any numerical cell of the domain can be expressed through the liquid phase fraction (α) as follows:

(6) $$\Upsilon =\alpha \Upsilon_{water}+\left(1-\alpha \right)\Upsilon_{oil}$$

where Υ stands for any physical properties involved in the problem, e.g., the density (ρ) and viscosity (μ) for both phases. In the case of polymer flooding simulation, water properties will be replaced by those of the polymer solutions with a shear-rate-dependent viscosity, $\mu (\dot{\gamma })$, as detailed in the next sub-section.

### 2.2. Fluid Rheology: Shear-Thinning Fluid

We propose a shear-thinning, power-law fluid, to model the non-Newtonian nature of the viscosity of the liquid, expressed as follows:

(7) $$\mu_{p}=\chi {\dot{\gamma }}^{n-1}$$

where χ and *n* are the consistency factor and the flow index, respectively. As a result Equation (6) can be expressed in terms of the viscosity as:

(8) $$\mu =\alpha \mu_{p}+\left(1-\alpha \right)\mu_{oil}$$

The power-law model is adopted to reflect the fluid employed in EOR polymer flooding, which exhibits shear thinning behavior in line with the work in [11]; the typical values are $\chi =\chi_{0}=10^{-2}$ Pa.s^*n*^ and n=0.81. The two-phase flow VOF model describes and discriminates the fluid in a given numerical cell through the phase fraction (α). Since α represents a mass fraction, varying between 0 and 1, Equation (8) assumes a linear relation between the amount of fluid in a cell and the average viscosity of the cell in line with the VOF framework [24,25,26,27,28]. However, Patankar [29] improves the accuracy in the case of non-uniform thermal conductivity. In the present case, the viscosity is uniform within each fluid defined by (α). In addition, the approach adopted regarding the viscosity has been validated for two-phase flow for both Newtonian and non-Newtonian fluids in [24,25,27,28,30]. In [26], the use of the arithmetic mean is adopted and validated for the viscosity while the harmonic average is employed for the thermal conductivity as suggested by Patankar [29].

Accounting for the viscosity, the stress tensor for a generalized Newtonian fluid can be written as:

(9) $$\tau =\mu_{p}\left(\gamma \right)\dot{\gamma }$$

where the rate-of-strain tensor expresses as,

(10) $$\dot{\gamma }=\nabla V+{\left(\nabla V\right)}^{T},$$

while the shear rate is given by:

(11) $$\dot{\gamma }=\sqrt{\frac{1}{2}(\dot{\gamma }:\dot{\gamma }})$$

### 2.3. Dynamic Contact Angle and Hysteresis Modeling: Fluid-Solid Matrix Interaction

**Figure 1:**
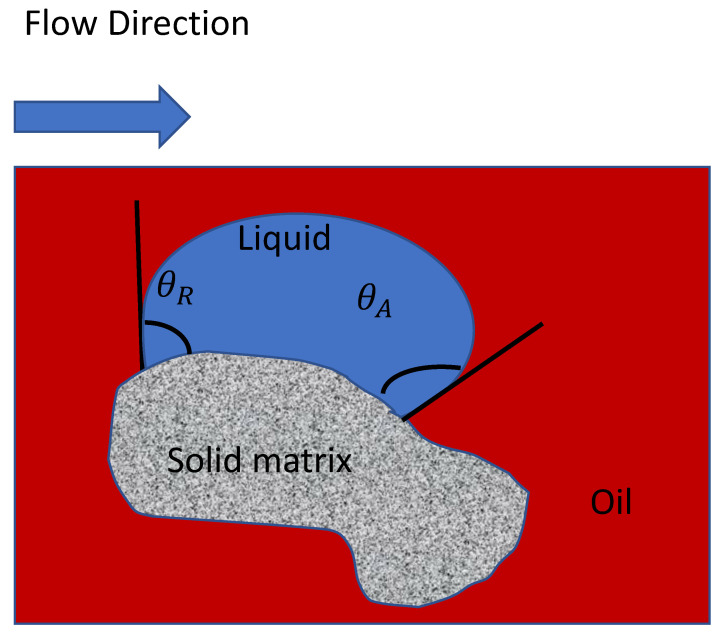
Schematic of advancing and receding contact angles of two-phase flow interacting with a porous solid matrix.

The interaction between the fluid and the solid matrix phase of the porous media is handled through the dynamic contact angle, which varies between the advancing ($\theta_{A}$) and receding ($\theta_{R}$) contact angles (Figure 1).

The accuracy of two-phase flow simulation is highly related to the way the dynamic contact angle is modeled. Here the dynamic contact angle ($\theta_{d}$) is implemented using the correlation by Kistler [31]:

(12) $$\theta_{d}=f_{H}\left[Ca+f_{H}^{-1}\left(\theta_{E}\right)\right]$$

where $Ca=\mu U_{cl}/\sigma$ corresponds to the capillary number, while $U_{cl}$ represents the contact line velocity, approximated by taking the velocity within the first cell above the solid phase. It is worth noting that, in contrast to the capillary number, Ca, adopted in [32] where it is based on the Newtonian fluid viscosity and derived from the dimensionless form of the governing equations; in the present work, however, Ca is defined based on the motion of the triple line velocity ($U_{cl}$) to model the dynamic contact angle in accordance with Kistler’s relation [24,25]. Since the shear-thinning fluid employed consists of dilute polymer in water, the contact angle between the fluid and the solid is mainly controlled by the physico-chemical properties of the solvent water. As a result, Ca used in in Equation (12) is based on the properties of water. Finally, the Hoffman function $f_{H}$ is given by:

(13) $$f_{H}\left(s\right)=\arccos\left\{1-2\tanh\left[5.16{\left[\frac{s}{1+1.31s^{0.99}}\right]}^{0.706}\right]\right\}$$

To model the contact angle hysteresis occurring when the fluid is moving on chemically or topologically heterogenous surfaces, the equilibrium or static contact angle given in Equation (12) is implemented with either the receding ($\theta_{R}$ ) or advancing ($\theta_{A}$) contact angle as per the direction of the triple line velocity of the oil-water-rock system. As a result, the model is able to simulate hysteresis of wettability at pore scale. As pointed out in Yokoi et al. [33], contact angle model played a key role in simulating the contact line motion of two-phase flow. It is worth noting that the approach adopted here was shown to accurately capture multiphase flow behavior under different configurations including both Newtonian and Newtonian fluids [24,30,34]. Finally, the contact angle at the solid surface is enforced to compute the capillary force in the momentum equation, Equation (3). The mean curvature required for the capillary effect is determined using the unit normal vector at the interface between the liquid and the solid as follows:

(14) $$n_{w}={\left(\frac{\nabla \alpha }{\left|\alpha \right|}\right)}_{w}=n_{n}\cos\left(\theta_{d}\right)+n_{t}\sin\left(\theta_{d}\right)$$

where $n_{n}$ and $n_{t}$ are the unit normal vector and tangential vector to the solid matrix, respectively. Further details can be found in [23,26].

### 2.4. Numerical Methods

**Figure 2:**
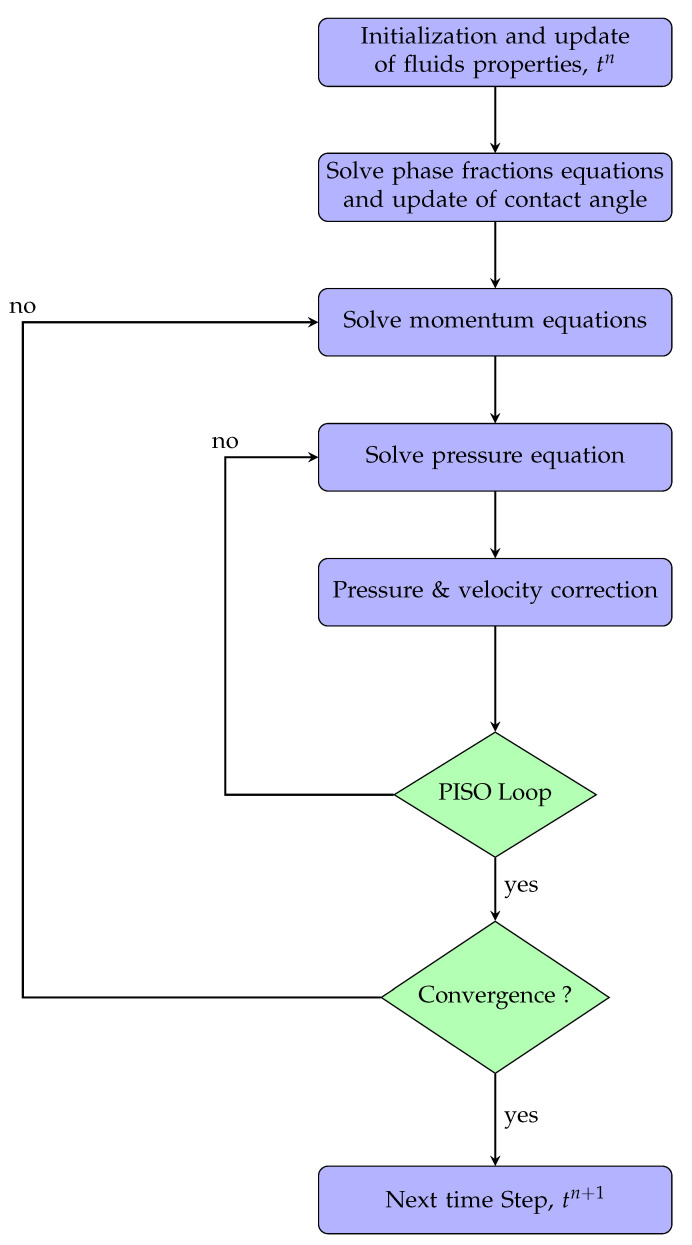
Numerical algorithm for solving the governing equations using the volume of fluid (VOF) method.

The governing equations for the two-phase flow simulation accounting for wetting hysteresis and fluid rheology are solved using Open-source Field Operation And Manipulation (OpenFOAM), a C++ toolbox for the implementation of numerical solvers. The Equations (1)–(14) are discretized by means of finite volume method (FVM) on an unstructured grid. FVM discretization are based on second-order and linear upwind-biased schemes. The two-phase flow governing equations within VOF framework are solved using a pressure implicit with splitting of operators (PISO) algorithm, coupled with a Gauss-Seidel smoother and generalized geometric-algebraic multi-grid (GAMG) solver. The numerical procedure is given in Figure 2. The details on the boundary conditions used are provided in Table 1.

The convergence of the iterative solver for the velocity and pressure fields are fixed at 10${}^{-6}$. Further details on the numerical methods can be found in our recent papers [11,24,30]. Because we want to investigate the wettability at both drainage and imbibition, all the simulations were performed until a steady state is reached. Then, the results are post-processed to compute both water and oil saturation.

## 3. Results and Discussion

### 3.1. Model Validation

#### 3.1.1. Single Phase Flow and 3D Rocks: Absolute Permeability

**Figure 3:**
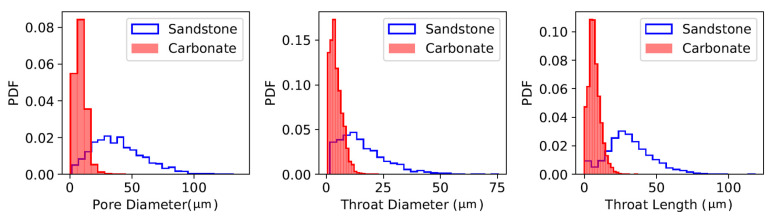
Statistics of the sandstone and carbonate pore network extracted.

To validate the approach based on FVM in porous media, numerical simulations to estimate the permeability are performed using a 3D micro-CT image of rock samples. The results obtained by FVM are subsequently compared to the values computed by both PNM and LBM. We consider two rock samples from the literature, the Fontainebleau sandstone and Grosmont carbonate [2], for the simulation of the three numerical techniques—PNM, FVM, and LBM. From the segmented images and to apply PNM, the pore network is extracted using the maximal ball algorithm [7,12]. Figure 3 depicts the statistics of the pore structure from the two samples in terms of pore diameter, throat diameter, and throat length distributions; as expected, the carbonate rock exhibits a smaller pore-network size compared to the sandstone sample spanning a relatively broad range of scales.

**Figure 4:**
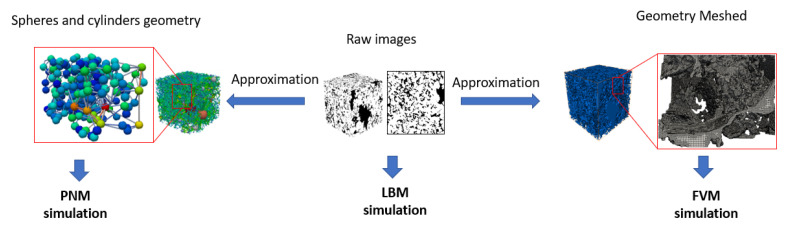
The input pore geometry for pore-network model (PNM), lattice Boltzmann method (LBM), and finite volume method (FVM) simulations based on the Grosmont carbonate micro-CT image.

The numerical simulation is performed on the two samples using the three numerical techniques. For illustration purposes, we show the approach for PNM, FVM and LBM simulations in Figure 4.

We summarize the results of the simulation of the permeability on the *z*-axis in Table 2. We can observe that FVM, which is based on VOF for single-phase simulation, yields better results compared to PNM and is within less than 3% compared to the more accurate LBM. However, in addition to being resource intensive, LBM relies on many adjusting parameters that make it less physically grounded to handle multiphase flow simulations compared to VOF. For these reasons, VOF is adopted in the present work and accounts for both fluid rheology and dynamic contact angle and hysteresis.

#### 3.1.2. The Relevance of Dynamic Contact Angle in Two-Phase Flow Modeling

**Figure 5:**
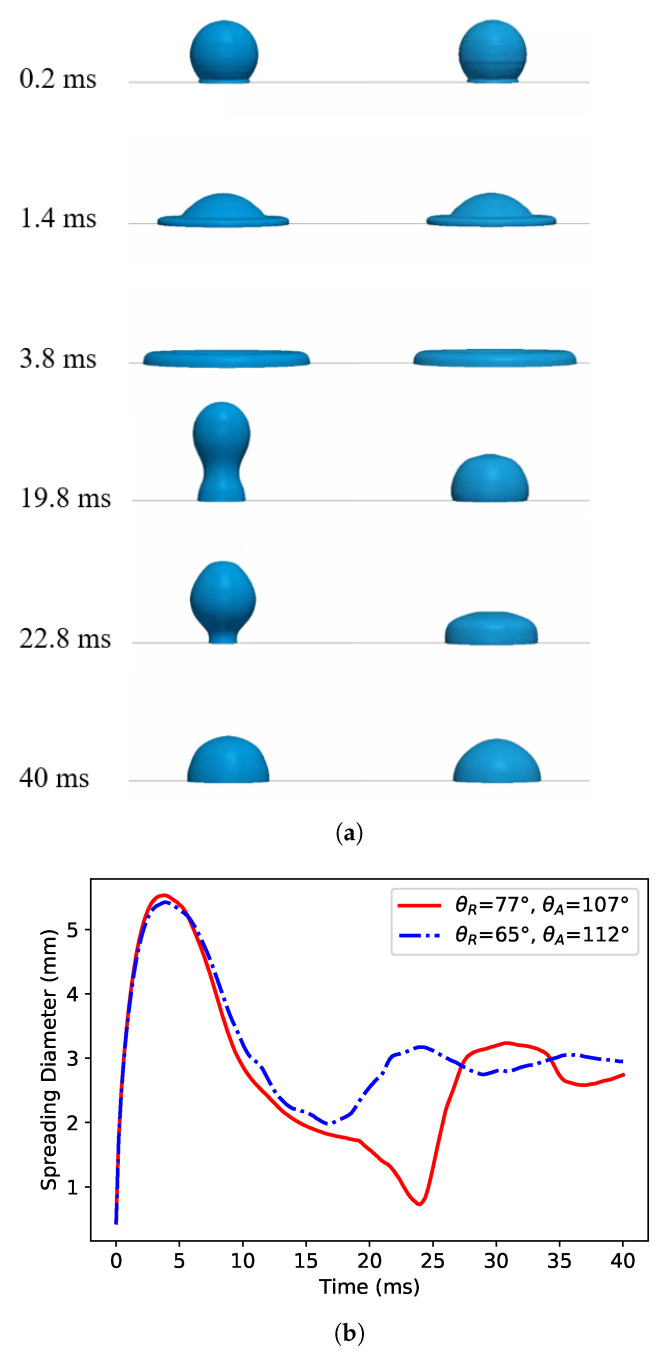
Effect of the contact angle hysteresis on droplet impact on a surface with the same equilibrium (or static) contact angle of *θ*_*E*_ = 90°, with droplet (**a**) snapshot and (**b**) spreading diameter. (**a**) Transient profile evolution for (left) *θ*_*R*_ = 77, *θ*_*A*_ = 107 and (right) *θ*_*R*_ = 65, *θ*_*A*_ = 112. (**b**) Spreading diameter evolution.

The accuracy of the two-phase flow modeling is highly related to the accurate description of the contact (or triple) line motion. Many studies use a static contact angle model instead of a dynamic model due to its simplicity. To validate and assess the relevance of our dynamic contact angle implementation, we analyzed the droplet dynamics upon impact on surfaces exhibiting contact angle hysteresis (CAH), which represent the difference between the advancing and receding contact angles, CAH=$\theta_{A}-\theta_{R}$. The impact of a water droplet on a surface is presented, and the effect of hysteresis is investigated. A comparison to the dynamics of a droplet of 2.3 mm impacting on the surface with the same static contact angle ($\theta_{E}=90^{\circ }$) but different contact angle hysteresis is carried out numerically. The result shows a stark contrast between the two configurations. An elongated droplet about to detach is observed in the low hysteresis case, while only a few oscillations of the droplet take place with the surface exhibiting a relatively high CAH, again with the same static contact angle (Figure 5). The result from the low CAH agrees with the results in [33,35]. This finding suggests that, for accurate modeling of multiphase flow, the dynamic contact description should be accounted for, and wettability through only the static contact angle is not sufficient.

**Figure 6:**
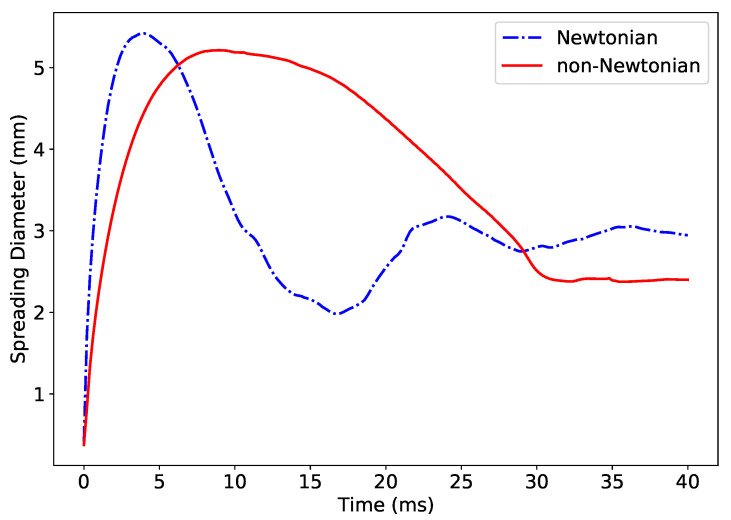
A comparative simulation of droplet impact of Newtonian and non-Newtonian fluids used in the present study.

In addition to the effect of the contact angle, we simulate the impact of the non-Newtonian droplet following a shear thinning power law as detailed in Equation (7). The evolution of the spreading diameter is shown in Figure 6. As expected, a contrast is observed for droplet impact dynamics behavior, with power low fluid exhibiting less oscillation upon impact before reaching equilibrium.

In the following, the developed VOF model will be employed to perform the simulations of both drainage and imbibition to gain a better physical insight into two-phase flow dynamics through porous media.

### 3.2. Two-Phase Flow in Porous Media with Hysteresis Effect

#### 3.2.1. Newtonian Fluid

The approach followed in the present work attempts to imitate the experimental setup for the characterization of two-phase, water and oil saturation through the steady-state method. For convenience, the two-phase flow simulation is performed here using a 2D geometry, and the liquids (oil and water) are considered to be Newtonian.

##### Effect of the Contact Angle Models

**Figure 7:**
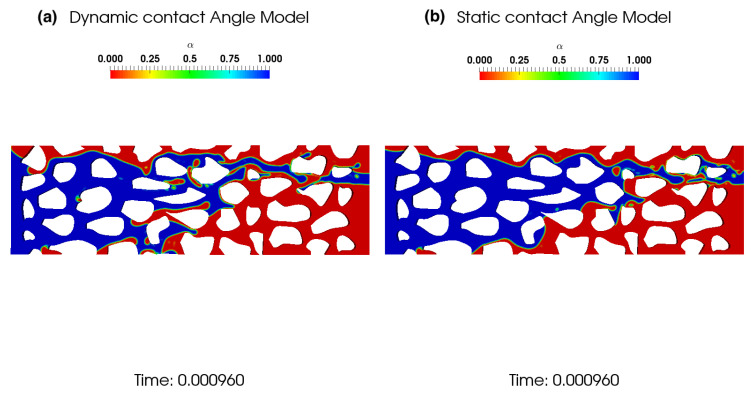
A comparative simulation of the effect of dynamic (**a**) and the static (**b**) contact angles.

In Figure 7, the effect of the contact on water saturation ($S_{w}$) within the porous media is investigated. We performed two implementations to assess the effect of both static and dynamic contact angles. The dynamic contact angle approach considers that during liquid motion, the contact angle, instead of maintaining a constant fixed value, varies following a dynamic contact angle following Kistler’s relation. We can observe that the fluid distribution is qualitatively different between the two configurations, even though they have the same equilibrium (or static) contact angle of $\theta =90^{\circ }$.

While the dynamic contact angle model is more physically grounded than the static one, this approach assumes the substrate to be ideal. In reality, substrates exhibit a wetting hysteresis effect, making the substrate prone to retain the liquid. This wetting effect will be detailed next.

We will focus on water saturation variation through a drainage test consisting of displacing the water phase by the oil to investigate the wettability effect on the fluid flow through porous media. Initially, the porous medium is 100% saturated with water ($S_{w}$ = 100%), and the primary drainage phase takes place by displacing water with oil. The injection of oil continues until no more water is eluted from the porous medium. The remaining water in place constitutes the irreducible water saturation ($S_{wi}$). It is worth noting that $S_{w}$ is the temporal variation of water saturation, which at steady state leads to $S_{wi}$.

**Figure 8:**
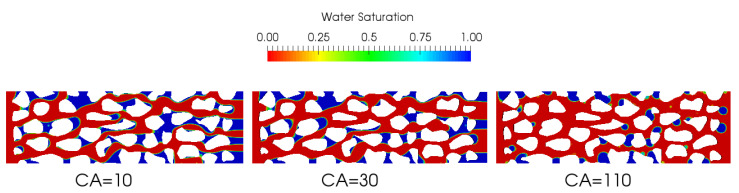
Effect of contact angle on water saturation.

Wettability plays a crucial role in the characterization of porous media. We show below the effect of contact angle on the fluid flow during the drainage phase, and the effect of the wettability will be assessed through $S_{wi}$. We implemented and simulated the configuration for three different contact angles: (i) static contact angle (SCA), (ii) dynamic contact angle (DCA), (iii) and dynamic contact angle with hysteresis (DCAH) effect. For the latter case, we consider a contact angle hysteresis of about 40${}^{\circ }$ in line with the standard deviation of the measured in-situ contact angle of actual carbonate rock [18]. In addition, we consider two configurations, namely water-wet SCA = 50${}^{\circ }$ and oil-wet SCA = 120${}^{\circ }$. We qualitatively illustrate the effect of the static contact angle on water saturation at $S_{wi}$ below in Figure 8. $S_{wi}$ is obtained by displacing water by oil until steady state, Figure 8 illustrates qualitavtively $S_{wi}$ at three static contact angles configurations. The water-wet configuration with a static contact angle of 10${}^{\circ }$ seems to trap more water compared to the two cases at 30${}^{\circ }$ and 110${}^{\circ }$. A quantitative analysis of these results are provided below in Figure 13.

**Figure 9:**
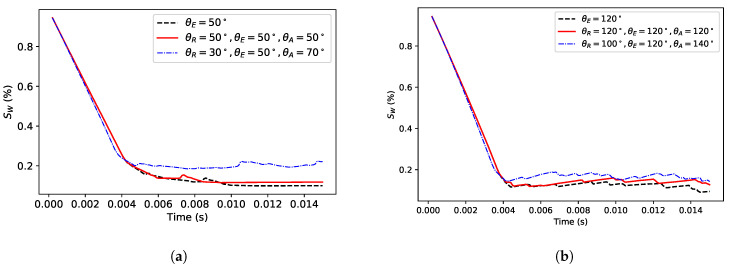
The evolution of the water saturation function of the contact angles under drainage for (**a**) water-wet and (**b**) oil-wet configurations, respectively.

The detailed temporal evolution of the water saturation profile function of the contact angle is shown in Figure 9. We can observe similar behavior among the three contact angle models at the early stage of the drainage, irrespective of the substrate wettability. However, the effect of hysteresis seems to be more pronounced under oil-wet conditions.

##### Effect of the Contact Angle Hysteresis

**Figure 10:**
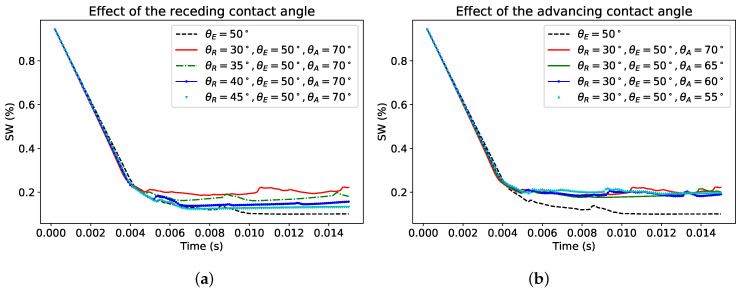
Sensitivity to the contact angles at the water-wet configuration for the receding (**a**) and advancing (**b**) contact angles.

To investigate the contact angle hysteresis, we consider two configurations of water-wet and oil-wet situations with a contact angle of 50${}^{\circ }$ and 120${}^{\circ }$, respectively. In each case, we vary the receding and advancing contact angles and compute the saturation ($S_{w}$). It is experimentally very challenging to perform such a sensitivity study on the effect of the advancing and receding contact angles. In the water-wet configuration, we obtain the result in Figure 10 by changing the advancing and receding contact angles numerically and maintaining all other parameters the same.

**Figure 11:**
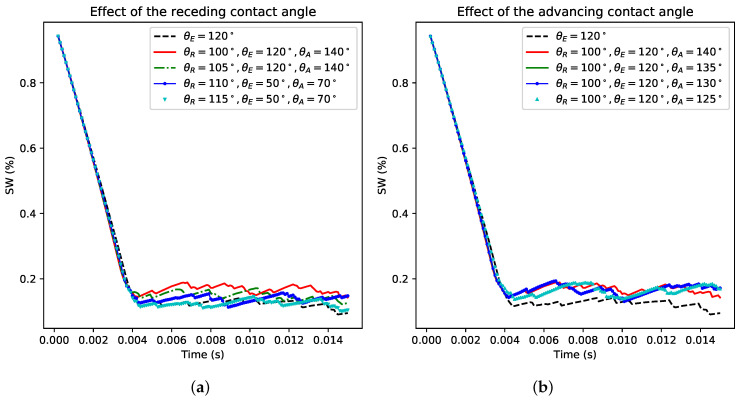
Sensitivity to the contact angles at the oil-wet configuration for the receding (**a**) and advancing (**b**) contact angles.

Similarly, for the oil-wet situation, with equilibrium contact of 120${}^{\circ }$, the sensitivity to both the receding and advancing contact angles are given in Figure 11 in terms of the temporal evolution of water saturation ($S_{w}$). While the effect seems limited on the overall saturation profiles, to gain better insight, we analyzed the influence on $S_{wi}$, which corresponds to the instant values of $S_{w}$ at the end of drainage process where there is no noticeable water production.

**Figure 12:**
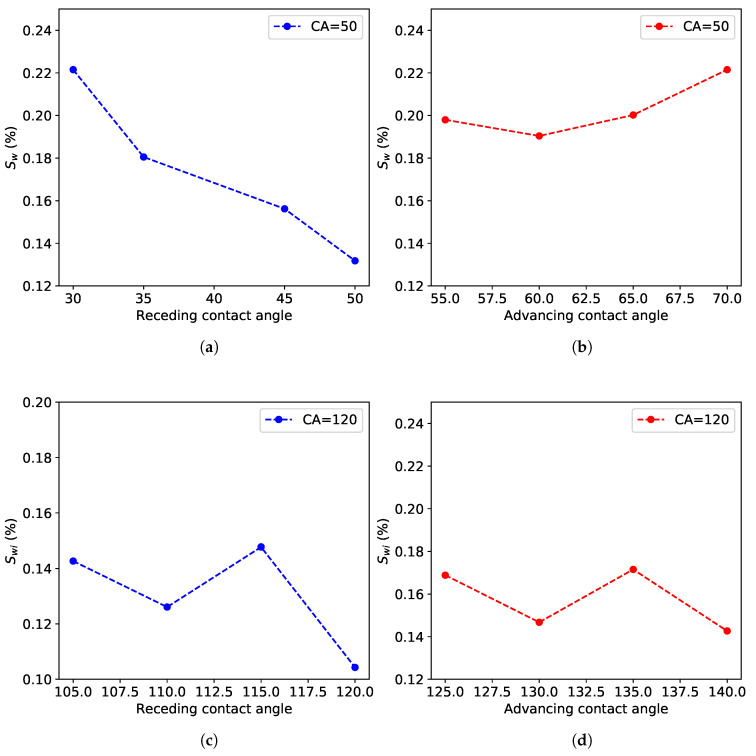
Sensitivity to receding and advancing contact angles in (**a,b**) water-wet and (**c,d**) oil-wet configurations.

We provide a summary of the sensitivity to the advancing and receding contact angles on $S_{wi}$ in Figure 12. The results indicate the key role played by the receding contact angle at both configuration water-wet and oil-wet. However, the water-wet system is more affected by the receding contact angle than the oil-wet. Interestingly, this result is in line with the prominent role played by the receding contact angle in controlling droplet contact time on impact on a superhydrophobic surface [36]. This finding can be valuable information to account for while formulating a strategy to produce more oil trapped in a porous medium. These results emphasize the capability of the model to perform such analysis at the pore scale, where a controlled experiment cannot be conducted to discern the role played by both the receding and advancing contact angles independently.

##### Effect of the Dynamic Contact Angle on $S_{wi}$ and $S_{or}$

**Figure 13:**
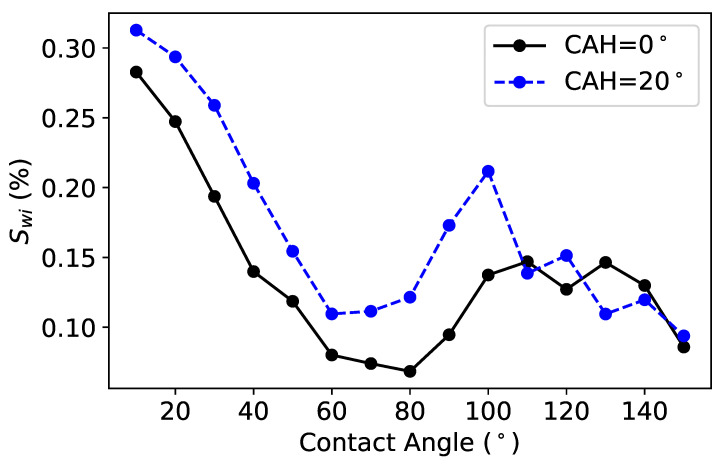
Effect of the wettability on $S_{wi}$.

While the contact angle is often assumed to be static for pore-scale modeling, we have estimated the effect of a dynamic contact angle on the fluid flow within a porous medium. We performed simulations with contact angles ranging from 10 to 150${}^{\circ }$. After examining the temporal variation of water saturation, we investigated the variation of the saturation as a function of the contact angle ranging from 10 to 150${}^{\circ }$, close to actual values in rock. The results in Figure 13 indicate the existence of an optimal value for the contact angle, minimizing $S_{wi}$. However, the simulations show that the relationship is much more complex, and the $S_{wi}$ function of wettability does not follow a monotonic relationship unlike the approximation assumed in the literature.

**Figure 14:**
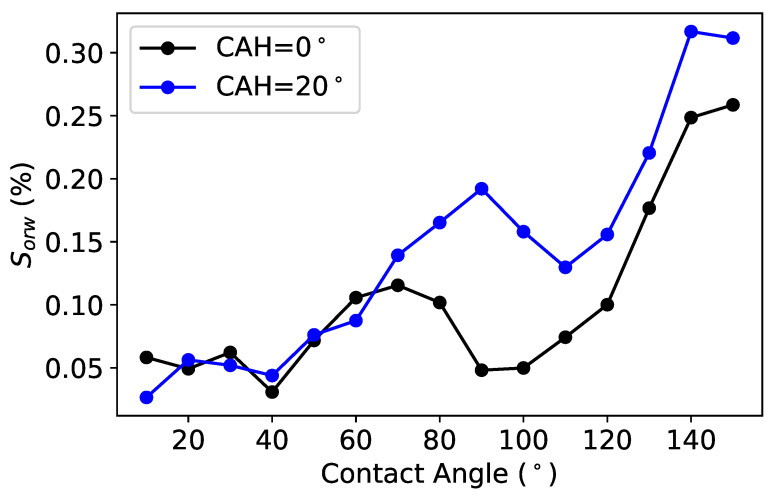
Effect of the wettability on $S_{or}$.

Similarly, following the drainage at $S_{wi}$, we performed a simulation of imbibition to track oil saturation ${(S}_{o})$, which is given by the ratio between the volume of oil and the volume of the pore space. We compute the residual oil saturation ($S_{or}$) at different contact angles ranging from 10 to 150${}^{\circ }$. The results of these simulations are shown below in Figure 14. Interestingly, the model indicates a minimum $S_{or}$ (or maximum recovery) in mixed–wet conditions when the contact angle is approximately 90${}^{\circ }$. However, the hysteresis effect, in which fluid tends to stick to surfaces, contributes to an increase in $S_{or}$, and thus decreases the potential recovery. Finally, $S_{or}$ increases at very high contact angles of approximately 150${}^{\circ }$, i.e., in strongly oil-wet conditions.

In the following discussion, we will investigate the combined effects of the dynamic contact angle and non-Newtonian fluid flow through porous media.

#### 3.2.2. Non-Newtonian Fluid

The non-Newtonian power-law liquid is adopted to reflect the fluid employed in EOR polymer flooding, which exhibits shear thinning behavior [11]. The sensitivity to the rheological parameters is not straightforward. For each values of *n* and *K*, the non-Newtonian simulation is performed for every contact angle ranging from 10 to 150${}^{\circ }$. A detailed simulation such as this on the effect of contact and its hysteresis is still to be addressed in the literature.

**Figure 15:**
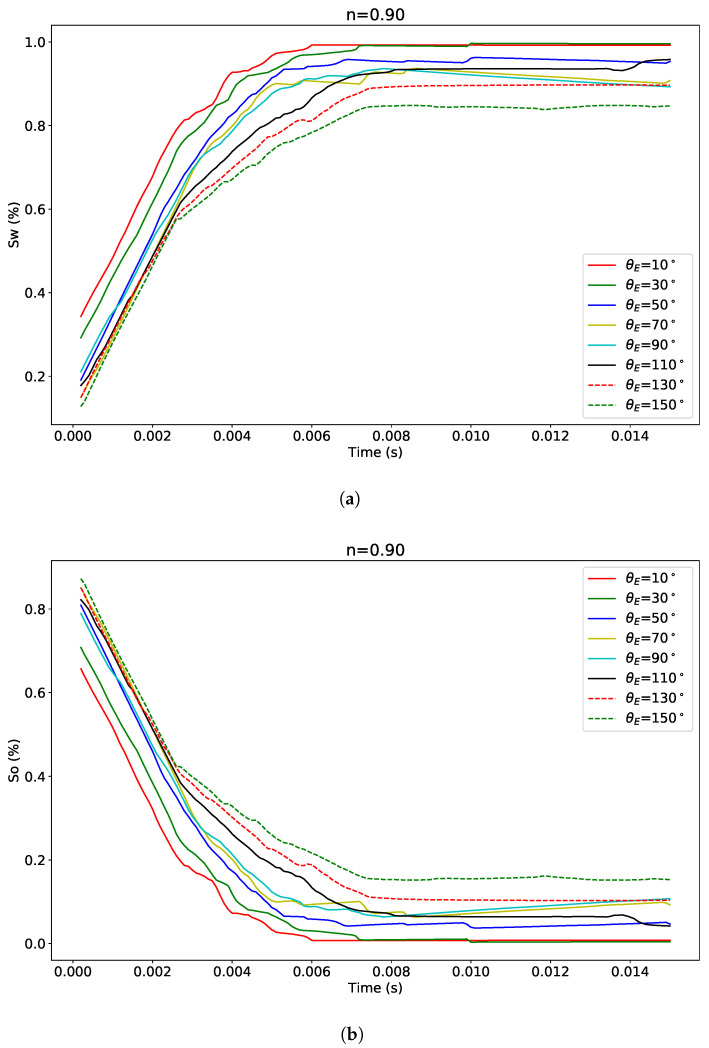
Temporal evolution of (**a**) polymer solution in water and (**b**) oil saturations at different contact angles for a non-Newtonian fluid, *n* = 0.9 and *χ* = *χ*_0_.

The effect of wettability on both polymer solution in water ($S_{w}$) and oil saturation ($S_{o}$) is investigated for a non-Newtonian fluid. We showed in Figure 15, the temporal variation of $S_{w}$ and $S_{o}$ at different contact angles; for clarity, the results are depicted for every 20${}^{\circ }$ instead of the full range at 10${}^{\circ }$ intervals. During the imbibition process, the polymer solution which is initially at $S_{wi}$ increases while oil is produced or displaced from the porous media, therefore ($S_{o}$) decreases until reaching $S_{or}$.

**Figure 16:**
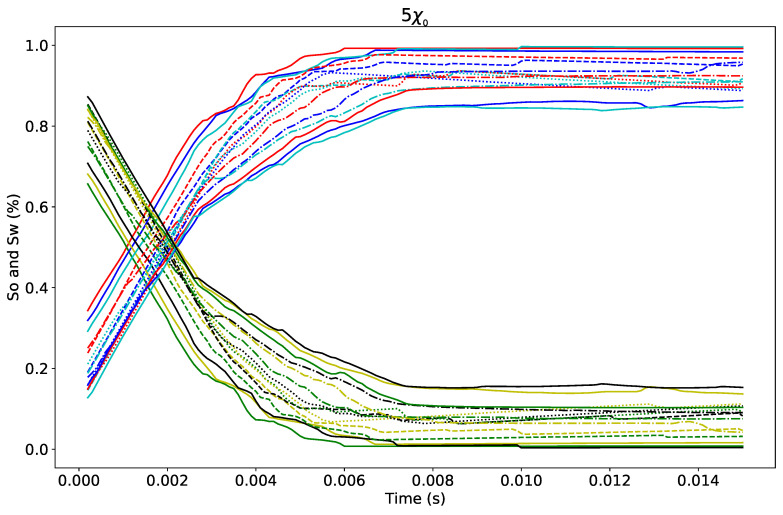
Effect of the wettability on both $S_{or}$ and Sw on non-Newtonian fluid with contact angle ranging from 10 to 150${}^{\circ }$.

Similarly, the simulation of polymer solution flowing through a porous media is shown at different contact angles with a consistency factor of $5\chi_{0}$. Figure 16 depicts the evolution of both polymer solution and oil saturations. The contact angle is varied between 10–150${}^{\circ }$ at every 10${}^{\circ }$. As in Figure 15, $S_{w}$ increases while $S_{or}$ decreases. A quantitative sensitivity analysis to both flow index and consistency factors is detailed below on residual oil saturation and wettability.

**Figure 17:**
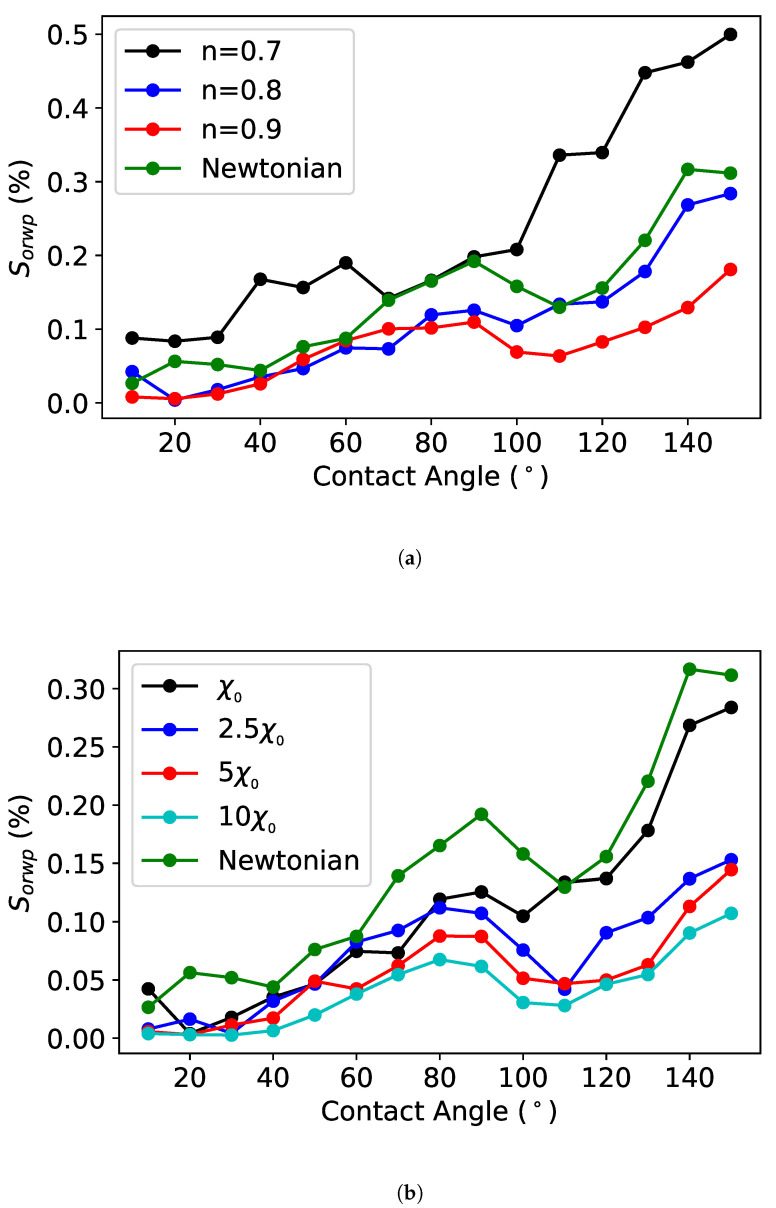
Sensitivity study on the flow index (**a**) and consistency factor (**b**) of the effect of the wettability on $S_{or}$ for a non-Newtonian fluid.

In order to perform a sensitivity analysis, we fixed all the parameters, and focused only on changing the values of the non-Newtonian properties such as the flow index and consistency factor at different contact angles. The governing equations are formulated and solved such that by taking n=1, χ=μ, we obtain the behavior of the Newtonian case. Therefore, the model can reliably capture the impact of the non-Newtonian nature of the flow through the pore space. To probe the wettability effect on the residual oil saturation, we changed the fluid rheological behavior, through n=1 and χ=μ, and analyzed their impact on the saturation evolution at the end of the imbibition process. As shown in Figure 17, we observed that increases in both *n* and χ lead to a decrease in $S_{or}$. It is worth noting that discriminating between the effects of the contributions of these two parameters to $S_{or}$ poses an experimental challenge. Therefore, the ability to perform such a sensitivity study numerically can aid in the formulation of polymer solutions for optimizing $S_{or}$.

## 4. Conclusions

We developed a volume of fluid (VOF) method to simulate a pore-scale model that takes into account the dynamic contact angle and its hysteresis. The two-phase modeling is based on the flow of both Newtonian and non-Newtonian fluids through a porous medium. The simulations are performed for drainage, in which water is displaced by oil until steady state is reached, followed by imbibition, in which oil is displaced by water or a polymer solution, leading to $S_{or}$. The physically based nature of the model and its accuracy enable us to study sensitivity to parameters that are not easily determined experimentally. The model is able to assess the effect of the contact angle and its hysteresis on both $S_{wi}$ and $S_{or}$. Interestingly, the model retrieves a maximum $S_{or}$ for mixed–wet configurations consistent with experimental observations. In addition, detailed simulations are performed to evaluate the influence of both the receding and advancing contact angles on two-phase flow in porous media. Finally, a pore-scale simulation mimicking polymer flooding is performed. Remarkably, the model is able to determine sensitivity to rheological parameters such as flow behavior index (*n*) and consistency factor (χ). The possibility of performing such a sensitivity study numerically can help in the formulation of polymer solutions for EOR.

## Figures and Tables

**Table 1:** Boundary conditions.

Boundaries	Pressures	Velocity	Phase Fraction (α)
Inlet	normal gradient, $\frac{\partial p}{\partial n}$ = 0	fixed valued, **V**	fixed value, α
Outlet	fixed value, p = 0 Pa	normal gradient,$\frac{\partial V}{\partial n}$ = 0	normal gradient, $\frac{\partial \alpha }{\partial n}$ = 0
Sides	normal gradient, $\frac{\partial p}{\partial n}$ = 0	fixed valued, **V** = 0	normal gradient, $\frac{\partial \alpha }{\partial n}$ = 0
liquid-solid interface	normal gradient, $\frac{\partial p}{\partial n}$ = 0	No-slip, **V** = 0	contact angle enforced through Equation (14)

**Table 2:** Numerical results of the two samples.

Sample Name	Voxel Size (μm)	Image Size (X × Y × Z)	PNM	FVM	LBM
Sandstone	7.5	288 × 288 × 300	1353	1614	1610
Carbonate	2.02	400 × 400 × 400	205	217	214
